# Head and neck cancers: a clinico-pathological profile and management challenges in a resource-limited setting

**DOI:** 10.1186/s13104-015-1773-9

**Published:** 2015-12-12

**Authors:** Japhet M. Gilyoma, Peter F. Rambau, Nestory Masalu, Neema M. Kayange, Phillipo L. Chalya

**Affiliations:** Department of Surgery, Catholic University of Health and Allied Sciences-Bugando, Mwanza, Tanzania; Department of Pathology, Catholic University of Health and Allied Sciences-Bugando, Mwanza, Tanzania; Department of Oncology, Catholic University of Health and Allied Sciences-Bugando, Mwanza, Tanzania; Department of Paediatrics, Catholic University of Health and Allied Sciences-Bugando, Mwanza, Tanzania

**Keywords:** Head and neck cancers, Clinicopathological, Challenges, Resource-limited setting, Tanzania

## Abstract

**Background:**

Head and neck cancer (HNC) is one of the most common cancers worldwide and its incidence is reported to be increasing in resource-limited countries. There is a paucity of published data regarding head and neck cancers in Tanzania, and Bugando Medical Centre in particular. This study describes the clinicopathological profile of HNC in our local setting and highlights the challenges in the management of this disease.

**Methods:**

This was a retrospective study of histopathologically confirmed cases of head and neck cancers treated at Bugando Medical Center between January 2009 and December 2013.

**Results:**

A total of 346 patients (M:F = 2.1:1) were studied representing 9.5 % of all malignancies. The median age of patients was 42 years. Cigarette smoking (76.6 %) and heavy alcohol consumption (69.9 %) were the most frequently identified risk factors for head and neck cancer. The majority of patients (95.9 %) presented late with advanced stages. Twenty-five (7.2 %) patients were HIV positive with a median CD4+ count of 244 cells/μl. The oral cavity (37.3 %) was the most frequent anatomical site affected. The most common histopathological type was carcinomas (59.6 %) of which 75.7 % were squamous cell carcinoma. A total of 196 (56.6 %) patients underwent surgical procedures for HNC. Radiotherapy and chemotherapy was reported in 9.5 and 16.8 % of patients, respectively. Only 2 (0.6 %) patients received chemo-radiation therapy. The mortality rate was 24.4 %. The overall 5-year survival rate (5-YSR) was 20.6 %. The predictors of overall 5-YSR were age of patient at diagnosis, stage of disease, extent of lymph node involvement, HIV seropositivity and CD4+ count <200 cells/μl (P < 0.001). Local recurrence was reported in 22 (23.4 %) patients and this was significantly associated with positive resection margins, stage of the tumor and presence of metastasis at diagnosis and non-adherence to adjuvant therapy (P < 0.001).

**Conclusion:**

Head and neck cancers are not uncommon at Bugando Medical Centre and show a trend towards a relative young age at diagnosis and the majority of patients present late with advanced stage cancer. Therefore, public enlightenment, early diagnosis, and effective cost-effective treatment and follow-up are urgently needed to improve outcomes of these patients in our environment.

## Background

Head and neck cancers are malignant neoplasms occurring in the nasal cavities, paranasal sinuses, nasopharynx, hypopharynx, oropharynx, ear, scalp, oral cavity and salivary glands [1]. They constitute a major public health concern throughout the world and are an important cause of morbidity and mortality [1–3]. Head and neck cancers are associated with high morbidity because there is interference with vital functions of life such as breathing, swallowing, speech, hearing, vision, taste and smelling [2].

Epidemiologically, head and neck cancers have been reported to be the tenth most common cancer worldwide [3] and there has been a significant increase in the global incidence of head and neck cancer over the past decade [4, 5]. At present, more than 650,000 new cases of head and neck cancer are diagnosed each year worldwide [5], and their incidence appears to be increasing in developing countries [4]. Globally, head and neck cancers constitute 5–50 % of all cancers [6], and 5–8 % of total body cancers in Europe and America [7–9], whilst in India it forms about 30 % of all cancers [10, 11]. Overall, head and neck cancers seem to affect black Africans at a younger age than in Caucasians [11]. Men are affected far more often than women [10, 11].

Etiological factors determining individual susceptibility to head and neck cancers are still not fully known [2, 12–14]. The pattern of occurrence of head and neck cancers varies between races and from one geographical area to another [2, 13]. Excess tobacco and alcohol consumption are the most important of the known predisposing factors [7]. The association of these predisposing factors with head and neck cancers makes these cancers preventable and controllable following early diagnosis [13, 14].

Diverse histological types of tumors are found in the head and neck region. More than 90 % of head and neck cancers are of epithelial origin, of which squamous cell carcinoma constitutes the greatest majority [15, 16]. Other histological types include lymphomas, blastomas, sarcomas and neuroendocrine tumors [16].

The management of head and neck cancer is complex and requires a multidisciplinary approach involving medical oncologists, radiation oncologists, head and neck surgeons, radiologists, speech therapists, social workers, psychologists, plastic and/or reconstructive surgeons, dentists with particular interest and expertise in head and neck cancer [17]. However, the management of cancers of the head and neck cancers remains a major challenge to medical practitioners because of the varied nature of histological patterns (sites of origin, natural history, and varied treatment modalities involving extensive, delicate, and sometimes repeated surgeries, radiotherapy and chemotherapy) [2, 7, 16, 17]. Thus the disease places great strain on health resources [2]. This strain is even more apparent in developing countries, such as Tanzania, where the bulk of health bills are borne by individuals and families since health insurance is not available.

The treatment of head and neck cancers varies according to the primary site, tumor stage, patient treatment preference, and practitioner’s expertise. Neck lymph nodes approach depends on the primary site and extent of disease [18]. The clinical stage of the disease at diagnosis often determines the prognosis and survival rate of a patient with head and neck cancers, with the best outcomes seen in patients diagnosed at an early stage [15, 16]. However, the outcome of treatment of head and neck cancers in limited-resource countries such as Tanzania has been poor because the majority of these patients present late to the hospital with an advanced stage of the disease and only palliative care is possible [19]. This is partly due to a lack of community awareness on the importance of early reporting to hospital for the early diagnosis and treatment of this condition, poor accessibility to healthcare facilities, limited diagnostic and therapeutic facilities, poor accessibility to adjuvant therapy and the high cost of care.

There is a paucity of information regarding head and neck cancer in Tanzania and Bugando Medical Centre in particular. This is partly due to a lack of published local data regarding this condition and the lack of cancer registries in this region. This study was designed to describe the clinicopathological pattern of head and neck cancer and highlight the challenging problem in the management of this disease in our local setting.

## Methods

This was a retrospective study of histopathologically confirmed cases of head and neck cancer treated at Bugando Medical Center between January 2009 and December 2013. Bugando Medical Centre is a consultant, tertiary care and teaching hospital for the Catholic University of Health and allied Sciences-Bugando (CUHAS-Bugando) and has a bed capacity of 1000. It serves as a referral center for tertiary specialist care for a catchment population of approximately 13 million people. The hospital has a newly established oncology department which provides care for all patients with histopathologically-proven cancers, including head and neck cancers. However, the department does not provide radiotherapy services at the moment due to lack of this facility at our center. As a result, patients requiring this modality of treatment have to travel long distances to receive radiotherapy at the Tanzania Oncology Centre located a considerable distance from the study area.

The study included all patients who presented to Bugando Medical Centre with histologically-confirmed cases of head and neck cancers during the period studied. Patients with incomplete data were excluded from the study.

The details of patients were retrieved from patients’ files kept in the medical record department, the surgical wards, operating theater and histopathology laboratory. Information retrieved included socio-demographic data, clinical presentation, anatomical site, tumor stage, histopathological type and grade, presence of metastasis (nodal, distant), HIV status, treatment modalities, and outcome and follow-up. HIV testing was performed using the Tanzania HIV Rapid Test Algorithm and CD4+ count using FACS or FACSCALIBUR (BD Biosciences, USA). A determination of CD4+ count was only performed in HIV-positive patients. Using CD4 cell counts severe immune suppression was defined by patients with CD4 200 cells per μl whilst moderate immune suppression is formed by patients with CD4 >200 cells per μl. Head and neck cancer was diagnosed based on a histopathological examination. The clinical stage of the disease was assigned to each patient by using TNM (AJCC cancer staging manual); this is a staging system which is an expression of the anatomical extent of the disease based on the extent of the primary tumor (T), absence or presence of and extent of regional lymph node metastasis (N) and absence or presence of distant metastasis. Depending on the site, biopsy specimens were obtained by excisional, incisional, curettage or punch biopsies. Tissue specimens were submitted for histopathological examination. Frozen section study was not available during the period of the study.

Treatment modalities included surgery, chemotherapy and radiotherapy. Patients were followed up for up to 5 years or death. Survival analysis was carried out with survival defined as the time between the date of commencement of treatment and the date of last follow-up or death. The recurrence of disease was confirmed by physical findings, radiological studies, endoscopic examination with biopsy and surgery.

### Statistical analysis

Data collected were analyzed using SPSS computer software version 17.0 (SPSS, Inc., Chicago, IL, USA). Data were summarized in the form of proportions and frequency tables for categorical variables. The median (and IQR) and ranges were calculated for continuous variables, whereas proportions and frequency tables were used to summarize categorical variables. The Chi square (χ^2^) test was used to test for the significance of association between the independent (predictor) and dependent (outcome) variables in the categorical variables. The level of significance was considered as P < 0.05. Multivariate logistic regression analysis was used to determine predictor variables that predicted the outcome.

### Ethical consideration

Ethical approval to conduct the study was obtained from the CUHAS-Bugando/Bugando Medical Centre joint institutional ethic review committee before the commencement of the study.

## Results

Out of 4011 patients who were registered with malignancies at Bugando Medical Center during the 5-year period, 382 were cases of head and neck cancers representing 9.5 % of all malignancies. Out of these, 346 of them constituting 90.6 % had documentary evidence of a confirmed histological diagnosis. Hence, subsequent data analysis was based on the 346 histologically confirmed cases. The age of patients at diagnosis ranged from 3 to 82 years with a median age of 42 years (interquartile range 40–46 years). The modal age group at presentation was 41–50 years accounting for 28.9 % of cases. Two hundred and forty-one (69.7 %) patients were aged 40 years and above. Out of the 346 patients, 234 (67.6 %) were males and 112 (32.4 %) were females with a male to female ratio of 2.1:1. The majority of patients, 289 (83.5 %) were mainly farmers from the rural areas located a considerable distance from Mwanza City. Most patients, 214 (61.8 %), had either primary or no formal education and more than three-quarters of them were unemployed. More than 90 % of the study patients had no identifiable health insurance.

The duration between disease onset and presentation for all the patients ranged from 3 to 24 months with 104 (30.1 %) patients presenting within 6 months of onset of illness and 242 (69.9 %) patients after 6 months. Two hundred-fourteen (61.8 %) patients visited the herbalist in the course of their illness before presenting to the hospital. Smoking and alcohol consumption were reported by 265 (76.6 %) and 242 (69.9 %) patients, respectively. Smoking and alcohol consumption habit were more common in male patients than in females and this differences were statistically significant (P < 0.001).

The oral cavity was the most frequent anatomical site for the head and neck cancers accounting for 37.3 % of patients (Table 1). TNM staging was documented in only 148 (42.8 %) patients. Of this, only 6 (4.1 %) patients were identified as being in early stages (TNM stage I–II) and 142 (95.9 %) patients were presented in advanced stages (stage III–IV). Lymph node involvement at the time of diagnosis was recorded in 102 (29.5 %) patients of which 34 (33.3 %) presented with N1 nodal disease, 20 (19.6 %) with N2a nodal disease and 48 (47.1 %) with N2b nodal disease. Distant metastasis was recorded in 98 (28.3 %) patients. The most common histopathological type was carcinomas which afflicted 206 (59.6 %) patients. Out of the 206 carcinomas, 156 (75.7 %) were squamous cell carcinoma. Lymphomas ranked second in 88 (25.4 %) patients followed by sarcomas in 38 (11.0 %) and neuroendocrine tumors in 14 (4.0 %) patients.

**Table 1:** Distribution of patients according to anatomical tumor site

Anatomical tumor site	Frequency	Percentages
Oral cavity	129	37.3
Pharynx	56	16.2
Larynx	48	13.9
Nasal/paranasal sinuses	39	11.3
Neck region	37	10.7
Salivary glands	26	7.5
Scalp	7	2.0
Ears	4	1.2

Plain radiographs of the skull, neck and chest performed in 234 (67.6 %) patients revealed abnormalities in 112 (47.9 %) patients. Abdominal ultrasound done in 26 (7.5 %) revealed liver metastasis in 12 (46.2 %) patients. Computed tomography (CT scan) and magnetic resonance imaging (MRI) were not available, which challenged our management, as this limited the tumour staging. Complete blood count, hemoglobin levels and ESR were done in all patients. More than three quarter of the patients had hemoglobin levels less than 10.0 gm/dl and ESR in the first hour was found ranging between 12 and 80 mm/1 h. Serological investigations for HIV infection revealed that 25 (7.2 %) patients were HIV positive. Of these, 6 (24.0 %) patients were known cases on highly active anti-retroviral therapy (HAART) and the remaining 19 (76.0 %) patients were newly diagnosed patients. There was no statistically significant difference between HAART treatment and head and neck cancers (HNC) prevalence and outcome (p > 0.001). CD4+ count distribution among HIV positive patients ranged from 145 to 866 cells/μl with the median CD4+ count of 244 cells/μl. A total of eight (32.0 %) HIV patients had CD4+ count below 200 cells/μl and the remaining 17 (68.0 %) patients had CD4+ count of >200 cells/μl. The proportion of HIV infected patients was significantly higher in patients with lymphomas (12.5 %) and sarcomas (26.2 %) than in patients with carcinomas (1.9 %) and neuroendocrine tumors (0 %) (P < 0.001) (Table 2).

**Table 2:** Distribution of histopathological type according to HIV status

Histopathological type	HIV status		*p* value
	HIV seropositive (N/%)	HIV seronegative	
Carcinomas	4 (1.9)	202 (98.1)	0.674
Lymphomas	11 (12.5)	77 (87.5)	0.011*
Sarcomas	10 (26.3)	28 (73.7)	0.001*
Neuroendocrine tumors	0 (0)	14 (100)	–

* Indicates that it is statistically significant

A total of 196 (56.6 %) patients underwent surgical procedure of which tumor excision was the most common surgical procedure performed in 61.2 % of cases (Table 3). Radiotherapy was indicated in 130 (37.6 %) patients. Of these, only 33 (9.5 %) patients received this modality of treatment. Chemotherapy was prescribed in 76 (22.0 %) patients. However, only 58 (16.8 %) completed the treatment. Only 2 (0.6 %) patients received chemo-radiation therapy.

**Table 3:** Distribution of patients according to the type of surgical procedure performed (N = 196)

Surgical procedure performed	Frequency	Percentages
Tumor excision	120	61.2
Excisional/incisional/endoscopic biopsy	20	10.2
Paratidectomy	20	10.2
Thyroidectomy	10	5.1
Tracheostomy	9	4.6
Mandibulectomy	7	3.6
Submandibulectomy	6	3.1
Laryngectomy	4	2.0

In this study, 84 patients died in-hospital giving a mortality rate of 24.3 %. The commonest causes of deaths were advanced malignancy, complications of HIV/AIDS and sepsis. According to multivariate logistic regression analysis, advanced age (>65 years), late presentation (>6 months), HIV seropositivity, CD4+ count below 200 cells/μl, stage of disease, and presence of metastasis at the time of diagnosis were found to be the main predictors of mortality (P < 0.001).

Follow-up of patients among survivors (262) ranged from 3 to 62 months with a median of 24 months (interquartile range 20–26 months). At the end of the follow-up period, only 94 (35.9 %) patients of the survivors were available for follow-up and the remaining 168 (64.1 %) patients were lost to follow-up. Out of 94 patients who were available for follow-up, only 54 patients were alive and well at the end of 5 years, giving an overall 5-year survival rate of 20.6 %. The 5-year survival rate for patients with early disease was higher than for those with advanced disease (P = 0.016). The predictors of overall 5-year survival rate were age of patient at diagnosis (P = 0.012), stage of disease (P = 0.041), extent of lymph node involvement (P = 0.011), HIV seropositivity (P = 0.004), CD4+ count below 200 cells/μl (P = 0.001). Evidence of cancer recurrence was reported in 22 (23.4 %) patients. Positive resection margins, stage of the tumor and presence of metastasis at the time of diagnosis and non-adherence to adjuvant therapy were the main predictors of local recurrence (P < 0.001).

## Discussion

Malignant tumors of the head and neck constitute one of the most frequent malignancies worldwide with about half a million new cases diagnosed per year [5], and their incidence appears to be increasing in developing countries [4]. In this study, cancers of the head and neck accounted for 9.5 % of all malignancies which is comparable with the global incidence of 5–50 % [6]. Our figure is comparable with a figure of 10 % that was reported in Yemen [20], but higher than that reported in the USA (5 %) [7–9] and Kuwait (7.4 %) [20]. A high figure of 40 % was observed in some Asian countries [21]. This difference in the incidence of head and neck cancers in these studies may be explained in part by the differences in exposure to risk factors such as cigarette smoking and alcohol consumption, viruses, diet and familial risks [20, 21]. The incidences of these diseases are higher in regions of the world where tobacco use and alcohol consumption is high [20].

In this study, the majority of patients were in the fourth decades of life. This is in keeping with most previous reports from other African studies [12, 15, 16, 19]. Overall, head and neck cancers seem to affect Africans at a younger age than in Caucasians [2, 5, 12, 15, 16, 19, 20]. The reasons for higher percentage of occurrence in younger age group among African are not so clear but they may be connected to issues of race, genetics, poverty and behavioral practices. Shorter life expectancy in Africans compared with Caucasians, and earlier exposure to risk factors have also been speculated [2, 20].

The male to female ratio of 2.1:1 in this study, is in agreement with 1:1 to 2.3:1 reported by Lilly-Tariah et al. [12] in which a meta-analysis review of twenty-seven relevant published articles on head and neck cancers in Nigeria from 1968 to 2008 was undertaken, but differs from the slight female preponderance (1:1.02) observed by Ologe et al. [16] in Nigeria. The reason for the male predominance in these studies could be attributed to the fact that some of the habits that have been associated with the occurrence of head and neck cancer, such as smoking and use of alcoholic beverages, are strongly associated with male gender [22]. The male preponderance in this study may be explained by the high rate of cigarette smoking and alcohol consumption by men as compared to females in the Tanzanian society as shown in this study.

Head and neck cancer has been reported in most studies to be more prevalent in people with low socioeconomic status [12, 15, 16, 19, 20]. This finding is reflected in our study in which the majority of patients were farmers coming from rural areas located a considerable distance from the study area and most of them had either primary or no formal education and were unemployed. This observation has an implication on the accessibility of healthcare facilities and awareness of the disease.

In the present study, the majority of patients presented late with advanced stage of cancer which is in keeping with other studies in developing countries [15, 16, 19, 20]. Late presentation in these countries may be due to ignorance, poverty, poor access to health services, and patients consulting traditional healers and using traditional medicines. Late presentation of cases is an area of head and neck cancer care in our center that requires urgent attention. We could not establish the reasons for late presentation in this study, owing to its retrospective nature. Detecting primary cancer at an early stage contributes to improved chances for successful treatment and thus for survival.

The role of alcohol and tobacco in carcinogenesis of head and neck cancers is well documented [6, 12, 22, 23]. In our study, history of alcohol consumption and smoking was documented in 69.9 and 76.6 % of patients, respectively. Using alcohol and tobacco together increases the risk of developing head and neck cancers even more [7]. The joint effect of alcohol and smoking when consumed together are potentiated and the final relative risk is multiplied [6, 7]. More recent data from case–control studies and meta-analyses indicate that HPV-16 is an independent risk factor for oral and oropharyngeal carcinomas [21]. Human papilloma virus (HPV) status was not known as the facility for testing HPV was not available at our centre.

In this study, the oral cavity was the most frequent anatomical site for the head and neck cancers which is in agreement with other studies [5, 20], but at variance with a report on the overall pattern of head and neck cancers from Nigeria, in which nasopharynx, nose and larynx were the three most common sites [2, 16]. The nose and paranasal sinuses accounted for the highest number of cases followed by the nasopharynx and least being the scalp in a study done by Onotai and Nwogbo [24] in Nigeria. Onyango and Macharia [19] in Kenya reported the larynx as the most common site affected followed by the tongue. The reason for this anatomical difference among these countries is not clear but may have to do with geographical location and the socio-cultural practice of the people in that region [24].

As reported in a study by Donkor and Boateng [25] in Ghana, most of patients in this study presented with advanced stage (III–IV). The large number of patients with advanced stage at the time of diagnosis may be attributed to by the fact that the majority of patients in this study presented late to our health facility. The majority of patients in our environment, especially those in the rural areas lack the financial means to access modern health facilities due to high poverty level and this is further compounded by harmful traditional beliefs and practices which make them visit the herbalist for solutions to their health problems so that by the time they present to us, their tumors would have reached advanced stages and hence a poor outcome in management.

In this study, lymph node and distant metastases at the time of diagnosis were recorded in 29.5 and 28.3 % of cases, respectively. A similar metastatic pattern was reported by other authors [15, 16, 19]. High lymph node and distant metastases in this study is attributed to the late presentation in the majority of patients.

In agreement with the literature worldwide [26–28], the majority of head and neck cancer in this study was epithelial in origin (carcinomas) and was mostly squamous cell carcinoma. By contrast, Amusa et al. [29] in Nigeria found that lymphoma was the most frequently diagnosed head and neck cancer. The prevalence of cancers, especially carcinomas among farmers could be related to the long exposure to the sun and composition of some of the chemical fertilizers which may contain substances with carcinogenic risks [29].

Pretreatment workup of the head and neck cancers is important to decide on indication and extent of the treatment [30]. Histopathological investigation of the collected specimen is an essential part of work up as it provides a histological diagnosis enabling the commencement of early and proper treatment. Fine needle aspiration biopsy is preferable to open biopsy of a cervical lymph node for the reasons that there is no tumor spread, no inconvenient scar to distort future surgical intervention, no delay between diagnosis and treatment and its simplicity. When a diagnosis of malignancy cannot be made by needle biopsy, then an open biopsy can be done provided it can be followed by a frozen section and a concomitant definitive neck dissection if peroperative positive histological diagnosis is obtained [31]. Open cervical lymph node biopsy can alter patterns of lymphatic drainage for up to 1 year following surgery [32] and creates a scar which distorts future surgical intervention therefore altering the outcome of treatment [31, 32]. Facilities for frozen section are not available in our center and delay in getting histopathology results from the open cervical biopsies and also following panendoscopy as seen in our study further compounds our patients’ problems as their tumors and disease process progresses further with eventual poor outcome. Accurate staging at the time of the diagnosis of head and neck cancer is critical for selection of the appropriate treatment strategy. Therefore, optimizing pre-treatment imaging in the diagnostic work-up is of great importance. CT and MRI are the corner stones of diagnostic work-up. Technical improvements will increase the value of these techniques even further. PET and PET-CT became a standard imaging techniques for head and neck patients. It may be helpful for the detection of occult primary tumours, but its sensitivity for the detection of occult lymph node metastases is too low. Alternatively, the sentinel node procedure may be sufficiently accurate to avoid elective treatment of the neck. Screening for distant metastases should be performed only in head and neck cancer patients with high risk factors by FDG-PET-CT [33]. However, CT scan and MRI were not available in this study, which challenged our management, as this limited the tumour staging.

In this study, HIV seroprevalence was found to be 7.2 %, a figure that is significantly higher than 5.3 % in the general population in Tanzania [34]. We could not establish the reason for the high HIV seroprevalence in our study. Failure to detect HIV infection during window period and exclusion of some patients from the study might have underestimated the prevalence of HIV infection among these patients. Patients with HIV infection have been reported to have a significantly higher risk of developing head and neck cancers [26]. The proportion of HIV infected patients in the current study was significantly higher in patients with lymphomas (12.5 %) and sarcomas (26.2 %) than in patients with carcinomas (1.9 %). The risks of several different cancer types are elevated in HIV-infected individuals due to behavioral and biological characteristics, immunodeficiency, and potentially chronic inflammation and immune dysfunction/senescence [26].

In the present study, the staging of head and neck cancers before definitive treatment was done according to the TNM (tumour, node, and metastasis) system thorough clinical evaluation and work up. However, one of the salient and challenging outcomes of this analysis is the poor levels of staging for cancer. Most of the cancers were poorly staged or the records could not be verified. However, since the records indicated only the clinical staging or TNM system, it was possible that most of the unknown were merely late presentations either at stages III and IV. However, the unusually large percentage of head and neck cancers with no clinical stage indicated in the healthcare records calls for urgent remedial action by all concerned parties.

Surgery, radiotherapy and chemotherapy either alone or in combination are the standard modalities of treatment of head and neck cancers [30, 35, 36]. The histologic cell type and the stage of the disease determine the choice of treatment. Lymphomas respond satisfactorily to chemotherapy. Early stages of head and neck carcinomas can be treated satisfactorily (cured) with radiotherapy alone or surgery alone. Stages III and IV carcinomas require a combination of salvage surgery and radiotherapy [36]. Radiotherapy may be used before or after surgery [23, 30]. Chemotherapy is also useful in cases of metastasis and recurrence. Chemotherapy may also be used for induction chemotherapy before radiotherapy [36]. Frozen section is a standard procedure to ascertain safety or otherwise of tumour margins in most centres around the world. In Tanzania, frozen sections are not routinely done in the management of head and neck cancer. In this study, surgery given either alone or in combination with radiotherapy and chemotherapy were performed in more than half of patients. The reason for the low rate of head and neck cancer resection in our series may be explained by the fact that the majority of patients presented late with inoperable tumors at the time of diagnosis, for which only palliative treatment was possible. Only 4.1 % of the patients in our series had head and neck cancer resection with curative intent which is in agreement with other studies in developing countries [12, 35, 36]. Radiation therapy has been shown to improve local control rates in locally advanced head and neck cancers [36]. However, in our study only 9.5 % of patients who required radiotherapy had access to this form of treatment. This concurs with other studies in resource-limited countries [35, 36]. Failure to access this modality of treatment in our patients can be explained by the fact that radiotherapy is not available in our center and therefore patients requiring this form of treatment had to travel long distances to receive radiotherapy at the only oncological center in the country. This sad observation calls for urgent establishment of radiotherapy services in our center. In the present study, only 16.8 % of patients received chemotherapy despite the establishment of an oncological unit in our center in 2009. In resource poor countries such as Tanzania, non-adherence to chemotherapy is a major challenge in cancer treatment including head and neck cancers. Reasons for non-adherence in most developing countries include financial difficulty, resorting to alternative treatment and drug side effects [12, 36]. We could not establish the reasons for non-adherence to chemotherapy in our study owning to the retrospective nature of the study. Further prospective study is needed to explain this observation. In developed countries, chemoradiation is widely used as an organ-sparing treatment strategy in patients with head and neck cancers. Compared to radiotherapy alone it has an 8 % advantage in terms of locoregional control and survival rates [35]. In this study, only 2 (0.6 %) patients who had squamous cell carcinoma of the oral cavity and nasopharynx received chemoradiation therapy at the Tanzanian Oncological centre located a considerable distance from the study area.

The overall mortality rate in this study was 24.3 %, a figure which is higher than 14 % reported by Kumar et al. [37], but low compared with 32.6 % that was reported by Adeyi and Olugbenga [35] in Nigeria. The commonest causes of deaths were advanced malignancy, complications of HIV/AIDS and sepsis. The high mortality rate in this study is attributed to advanced age at diagnosis, late presentation, HIV seropositivity, CD4+ count below 200 cells/μl, stage of the cancer and presence of metastasis at the time of diagnosis. Addressing these factors responsible for high mortality in our patients is mandatory to be able to reduce mortality associated with this disease.

In keeping with other authors in developing countries [12, 35, 38], the follow-up of patients in this study was generally poor as more than 60 percent of patients were lost to follow-up at the end of 5 years. We could not establish the reasons for the large number of loss to follow up in this study. This calls for further studies to explain this state of affairs.

Despite recent advances in the diagnosis and treatment of head and neck cancer, there has been little evidence of improvement in 5-year survival rates over the last few decades [35, 38]. In this study, the overall 5-year survival rate of 20.6 % is significantly low compared to the survival rate for patients with advanced (stage III–IV) head and neck cancers managed in developed countries which ranges from 30 to 50 percent [19, 35, 38]. The lower survival rate in this study is consistent with data from most cancer sites in developing countries and is likely to be due to late diagnosis, late referrals, poor access of patients to our centre and the reliability on traditional healers and medicine man [35]. In our patients, the factors that significantly affect prognosis were age of patient at diagnosis, stage of disease, extent of lymph node involvement, HIV seropositivity and CD4+ count below 200 cells/μl.

Despite advances in the treatment of head and neck cancer, 15–50 percent of patients will develop recurrent disease [39]. In this study, local recurrence of cancer was reported in 23.4 % of cases, a figure which is higher than that reported in other studies [12, 19, 35]. The reasons for the high rate of local recurrence in the present study may be attributed to advanced stage of cancer at the time of diagnosis, presence of metastasis at the time of diagnosis, positive resection margins and non-adherence to adjuvant therapy.

This study has demonstrated that the management of head and neck cancers poses diagnostic and therapeutic challenges at Bugando Medical Centre and contributes significantly to high morbidity and mortality among these patients. Factors such as late presentation, inaccessible health facilities and limited diagnostic and therapeutic tools have contributed to the eventual poor outcome in the management of patients with head and neck malignancies in our environment. Delay in the availability of histopathology results following biopsies and the practice of subjecting patients with head and neck lymphadenopathy to open lymph node biopsy with attendant complications such as scar formation and tumor spread are the hallmarks of the disease in this part of the world [35].

The major limitation of this study is the fact that information about some patients was incomplete in view of the retrospective nature of the study and poor documentation might have introduced some bias in our findings. Also, this study included only patients who were evaluated and treated at a single institution, which may not reflect the whole population in this region, despite the fact that approximately seventy percent of oncologic patients in northwestern Tanzania are managed at our center. Lack of advanced staging investigations such as CT scan and MRI challenged our management, as this limited the tumour staging. In addition, follow-up was poor and irregular and so it was difficult to know the exact time of recurrence or death. Large number of loss to follow up was also a potential limitation of this study as this may have underestimated the local recurrence and overall survival rate. However, despite these limitations, the study has provided local data that can help healthcare providers in the management of patients with head and neck cancer. The challenges identified in the management of head and neck cancer in our setting need to be addressed in order to deliver optimal care for these patients.

## Conclusion

Head and neck cancers are not uncommon at Bugando Medical Centre and show a trend towards a relative young age at diagnosis and the majority of patients present late with advanced stage cancer. Lack of awareness of the disease, poor accessibility to healthcare facilities, limited diagnostic and therapeutic facilities, the delay in the availability of histopathology results following biopsies, poor accessibility to adjuvant therapy, the high cost of care, a high morbidity and mortality, poor follow up and loss to follow up are among the hallmarks of the disease in this region and pose a great challenge in the management of these patients. Therefore, public enlightenment, early diagnosis, and effective cost-effective treatment and follow-up are urgently needed to improve outcomes of these patients in our environment. There is a need for the government to provide treatment funds for these poor patients as a significant number of patients were unable to complete treatment due to lack of funds. Establishment of radiotherapy services at our centre is highly recommended.

## Abbreviations

CUHAS: Catholic University of Health and Allied Sciences
HNC: head and neck cancer
TNM: primary tumor, regional lymph node, distant metastasis
CT: computed tomography
MRI: magnetic resonance imaging
